# Correlation between circulating interleukin-18 level and systemic lupus erythematosus: a meta-analysis

**DOI:** 10.1038/s41598-021-84170-4

**Published:** 2021-02-25

**Authors:** Mengmeng Xiang, Yang Feng, Yilun Wang, Jie Wang, Zhixiong Zhang, Jun Liang, Jinhua Xu

**Affiliations:** 1 Department of Dermatology, Huashan Hospital, Fudan University, Shanghai, 200040 China; 2 Cutaneous Biology Research Center and Melanoma Program MGH Cancer Center, Harvard Medical School/Massachusetts General Hospital, Boston, MA 02114 USA; 3 Shanghai Institute of Dermatology, Shanghai, 200040 China

**Keywords:** Systemic lupus erythematosus, Biomarkers

## Abstract

This study is a meta-analysis aimed at pooling reported data and clarifying the association between circulating level of interleukin-18 and systemic lupus erythematosus (SLE). We searched medical databases including Medline/Pubmed, Embase, Scopus, The Cochrane Library, and Web of Science thoroughly to obtain all related articles published before July 15th, 2020. We pooled computed standardized mean difference (SMD) and its 95% confidence interval using STATA 13.0 and exhibited in the form of forest graph. Meta-regression and subgroup analysis were also performed to explore the source of heterogeneity. Publication bias was first evaluated by the symmetry of the funnel plot and then Egger’s linear regression test. Thirty eligible studies from eighteen regions were finally included and the relevant data from these studies were pooled. The analysis results displayed that SLE patients showed a significantly higher level of circulating IL-18 level in comparison with healthy controls (SMD = 1.56, 95% CI [1.20–1.93]; I^2^ = 94.9%, *p* < 0.01). The conclusion was equally applicable in subgroups divided based on sample type, mean age, disease duration, and testing method. Patients with SLEDAI score higher than five, or who were Asian, White, Arab, or mixed ethnicity had an elevated level of IL-18, while the others didn’t. This meta-analysis has elucidated that compared with healthy people, the circulating level of IL-18 is considerably higher in SLE patients, which indicates the underlying role of IL-18 in SLE pathogenesis.

## Introduction

Systemic lupus erythematosus (SLE), recognized as an autoimmune disease, is inclined to involve a number of targeted organs like kidneys, lungs, and the nervous systems, which may cause immense damage in the process of abnormal inflammation^1^. The incidence of SLE is about 10–150 patients among one hundred thousand people, predominantly in females which show seven to ten times higher rate than males^2^. The underlying mechanism for SLE is still in exploration, till now which can be briefly characterized into several improper procedures of the immune system, including impaired self-tolerance to nuclear antigens, the production of pathogenic auto-antibodies, and harmful depositions of immune complexes in targeted organs^3^. Due to the limitations in clarifying the exact initiation and progression of SLE, effective and precise treatments with fewer side-effects have still lacked. Thus, an urgent need is coming up to deepen the understanding of SLE mechanism with the aim not only to move a step further in explaining pathogenesis comprehensively but also to provide meaningful and potential targets for following treatment^4^.

Interleukin-18 (IL-18), a pro-inflammatory cytokine, is categorized as a member of the interleukin-1 (IL-1) superfamily which shares several similar attributes in physiology functions with other IL-1 family members like IL-1α and IL-1β^5^. It can be released by dendritic cells, monocytes, macrophages, neutrophils, and epithelial cells and then, in turn, exert effects on immune cells affecting their cell survival, maturation and cytokine production. IL-18 binding with its receptor will recruit MyD88 and activate the NFĸB signaling pathway leading to the production of IFNγ, which brings about a TH1 type immune response^6^. IL-18 has hitherto been revealed to elevate in patient’s periphery blood in a slew of immune-related diseases including rheumatoid arthritis, asthma and inflammatory bowel disease^7–9^. Accumulating evidence has elucidated that IL-18 may also have a close relationship with SLE manifestations and play an underlying role in its pathogenesis. In MRL/lpr mice model, disease severity worsened when exogenous IL-18 was given whereas lupus was alleviated when mice were treated with anti-IL-18^10^. In SLE patients, levels of IL-18 in serum and skin were found higher than normal controls^11^. Besides, correlations between IL-18 and SLE severity, incidence of lupus nephritis had been pointed out^12^. We can therefore put forward the hypothesis that IL-18 has the potential to act as a promising biomarker in SLE.

Meta-analysis has widely acknowledged functions in alleviating study design differences across variant researches on the same topic and generating believable outcomes. Even though there are several studies that have been published to elaborate on IL-18’s function in SLE and correlations between polymorphisms of IL-18 and SLE have been confirmed, a meta-analysis with reliable quality exploring the correlation of circulating IL-18 and SLE is still needed^12,13^. We performed this meta-analysis aimed at pooling reported data and clarifying IL-18’s association in SLE patients.

## Methods and materials

### Searching strategies

We searched medical databases including Medline/Pubmed, Embase, Scopus, The Cochrane Library and Web of Science thoroughly to obtain all related articles published before 15th July 2020. Searching terms combined of medical subject headings (Mesh terms) “systemic lupus erythematosus”, “IL-18”, “serum”, “circulating”, “plasma” and their corresponding free words were applied. Strategies were adapted according to different searching requirements for previously mentioned databases. Detailed searching queries were provided in the Supplementary Table S1. Besides, reference lists for articles were also carefully screened in case of any omissions. All the literature retrieval procedures were carried out by two authors (Mengmeng Xiang and Feng Yang) independently. If the full text cannot be retrieved directly, we would contact study authors to get more information. All the procedures followed Preferred Reporting Items for Systematic Reviews and Meta-Analyses (PRISMA) and the checklist was uploaded as Supplementary Table S2.

### Inclusion and exclusion criteria

Articles which met following standards were going to be contained for further data processing: (1) patients diagnosed as SLE under a definite standard and matched healthy controls had been recruited; (2) circulating IL-18 level from SLE patients and healthy controls were measured and reported either in plasma or serum; (3) study types including the cohort, case control and cross-section; (4) the content was written in English. There were not any restrictions on ethnicity.

We had removed reviews, conference abstracts, editorials, commentaries, case reports and studies performed on non-humans for lack of required information. Studies had also been exempted if SLE patients were pregnant, infected or with other autoimmune diseases. As for repeated or duplicated studies, the more comprehensive and updated one would be selected.

### Data extraction and quality assessment

We reviewed all potentially eligible articles again in order to extract useful data and information, including author, region, year of publication, ethnicity, study design, number, mean age, gender, sample type, IL-18 circulating level of SLE patients and normal controls, measurement method, diagnostic standard of SLE, disease duration and SLEDAI score. Data was extracted directly if the study reported the mean and standard deviation of circulating IL-18 level. If values were presented in median, standard error, range and inter quartile range, they were converted into mean and standard deviation for analysis using previously reported formulae^14^. In the meantime, for the sake of evaluating study quality of each article, Newcastle–Ottawa-Scale (NOS) criteria were exploited for case–control studies and cohort studies^15^. The NOS criteria are composed of three assessing aspects, namely selection, comparability and outcome assessment with a highest score of nine. Agency for Health-care Research and Quality (AHRQ) cross-sectional study quality assessment which contained eleven elements was used for cross-sectional studies. If the aggregated NOS score or AHRQ assessment score is above six, the study quality can be regarded as relatively reliable. Two reviewers (YiLun Wang and Jie Wang) completed the extraction independently and the final consent was met in case of any disagreements with the intervention of another reviewer (Jun Liang).

### Statistical analysis

We obtained the mean and standard deviation of circulating IL-18 level from the included studies and then calculated the standardized mean difference (SMD) and its 95% confidence interval. All SMD values were pooled and presented in the form of forest plot. As for research heterogeneity, the Cochran’s Q statistic and Higgins I-squared statistical analysis (I^2^ = [(Q − df)/Q] × 100%) was undertaken^16^. If the *p* value is less than 0.05 or calculated I^2^ is over 50%, significant heterogeneity may exist and hereby a random-effect model would be used, or else the fixed-effect model ought to be utilized. Meta-regression and subgroup analysis were also performed to explore the source of heterogeneity. Publication bias was first evaluated by the symmetry of funnel plot and then Egger’s linear regression test was tried to assess more precisely^17^. If a significant publication bias was detected, the trim and fill method was conducted to yield an unbiased effect size through re-computing the probable missing studies^18^. What’s more, the sensitivity test was simultaneously made to ensure the stability of this meta-analysis. The entire data analysis process was completed using STATA 13.0 (STATA, College Station, TX). If the *p* value is under 0.05, it would be recognized as statistical significance.

## Results

### Basic features and quality assessment

**Figure 1:**
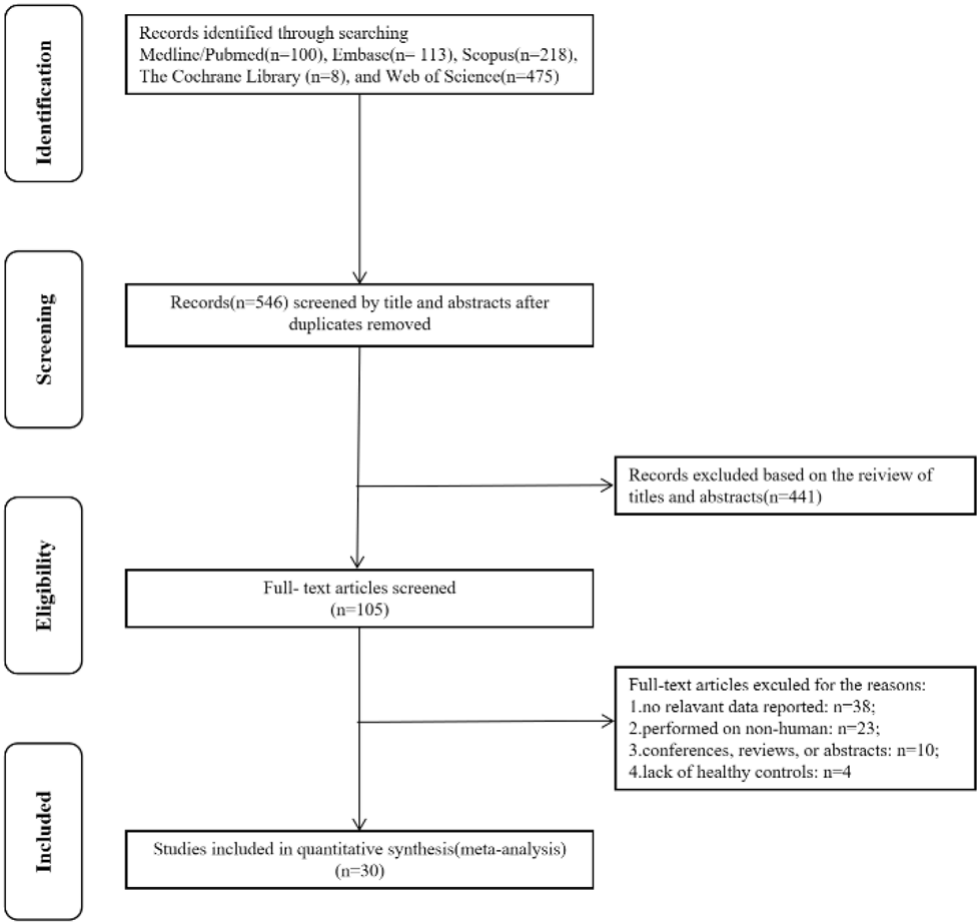
Flow chart of the inclusion and exclusion process.

Utilizing the search strategies listed above, a total of 914 studies were retrieved. 546 of them were then browsed by the title and abstract after removing 368 duplicates. Only 105 were scanned further by full text and eventually 30 of them were confirmed the eligibility for inclusion^11,12,19–46^. The flow chart of the detailed inclusion and exclusion process was displayed in Fig. 1. With sample sizes varying from 26 to 200, 1968 SLE patients and 1439 normal controls were taken under analysis. These studies were carried out from 2001 to 2019, in altogether 18 different regions. Among thirty included studies, six tested IL-18 in plasma, while the rest reported serum level. The methods employed to measure circulating IL-18 level included enzyme linked immunosorbent assay (ELISA) for twenty-six of them, cytokine multiplex assay for two studies, proximity extension immunoassay (PEA) and electrochemiluminescence (ECL) assay for one study separately. Almost all of them had used different versions of American College of Rheumatology (ACR) diagnostic standard for conformation of SLE patients. One study diagnosed under the Systemic Lupus International Collaborating Clinics (SLICC) classification criteria and another one followed Toronto SLE Disease Activity Index (SLE-DAI). All relevant information was listed in Table 1 including disease duration and SLEDAI score of SLE patients. As for study quality assessment, most of the included studies can be recognized as reliable quality. The score of each research for NOS scale or AHRQ assessment was listed in Table 1.

**Table 1:** Basic characteristics of included thirty studies.

Author	Year	Region	Sample type	Testing method	Study design	Study quality	Healthy controls			SLE patients					
							Size	Mean age (y)	Sex (F/M)	Size	Mean age (y)	Sex	Diagnose criteria	Disease duration (y)	SLEDAI score
Kawashima et al.^19^	2001	Japan	Serum	ELISA	CS	5	53	NP	NP	33	NP	NP	1982ACR	NP	> 8
Robak et al.^21^	2002	Poland	Serum	ELISA	CC	5	20	38 [18–65]	18/2	52	41 [17–76]	48/4	1982ACR	5.2 [0.08–23]	NP
Amerio et al.^20^	2002	Italy	Serum	ELISA	CS	5	20	NP	NP	20	19–59	18/2	1982ACR	NP	NP
Calvani et al.^22^	2004	USA	Serum	ELISA	CS	6	44	35.3 ± 14.6	39/5	72	42.1 ± 10.9	68/4	1997ACR	NP	NP
Park et al.^23^	2004	Korea	Serum	ELISA	CC	6	35	29.1 ± 9.4	33/2	35	29.7 ± 8.8	33/2	1997ACR	NP	19.8 ± 6.4
Tso et al.^24^	2006	Taiwan	Plasma	ELISA	CS	6	40	NP	40/0	72	38	72/0	1982ACR	9	4
Lit et al.^25^	2007	Hong Kong	Plasma	ELISA	CS	6	40	40 ± 9	39/1	80	37 ± 8.5	78/2	1982ACR	12.5 ± 6.2	5.5 ± 4.0
Xu et al.^26^	2007	Singapore	Plasma	ELISA	CS	5	113	NP	NP	76	NP	NP	ACR	NP	NP
Figueredo et al.^27^	2008	Brazil	Plasma	ELISA	CS	7	14	15.5 ± 1.5	0/14	16	15.6 ± 2.7	0/16	1997ACR	4 [1–10]	NP
Chen et al.^28^	2009	Taiwan	Serum	ELISA	CS	7	174	36.8 ± 12.4	85/89	165	32.3 ± 13.5	150/15	1997ACR	6.7 ± 4.3	15.4 ± 4.4
Lee et al.^29^	2009	Taiwan	Serum	ELISA	CC	6	30	NP	16/14	30	37.3 ± 16.3	27/3	1982&1997ACR	NP	27.8 ± 10.9
Novick et al.^31^	2010	Israel	Serum	ECL	CC	6	100	NP	NP	48	43.9 ± 16.3	38/10	1982ACR	NP	NP
Hu et al.^30^	2010	China	Serum	ELISA	CC	5	20	NP	NP	46	31.1 ± 11.7	38/8	1997ACR	NP	13.0 ± 5.6
Sahebari et al.^35^	2012	Iran	Serum	ELISA	CC	7	50	29 ± 7	NP	114	30 ± 9	NP	1997ACR	4	11.97 ± 10.07
Hermansen et al.^32^	2012	Denmark	Serum	Cytokine multiplex assay	CC	6	10	28 [24–48]	10/0	26	41 (19–70)	26/0	1982ACR	9.1 (0.1–30)	9 (0–20)
Koenig et al.^33^	2012	Switzerland	Serum	Cytokine multiplex assay	CS	6	14	38 (34–54)	11/3	26	38.76 ± 14.38	20/6	1982ACR	[0–24]	3.31 ± 3.02
Liu et al.^34^	2012	China	Serum	ELISA	CC	6	20	34.3 ± 11.4	17/3	46	31.1 ± 11.7	38/8	1997ACR	NP	13.0 ± 5.6
Aghdashi et al.^37^	2013	Iran	Serum	ELISA	CS	6	25	30.28 ± 5.53	25/0	50	32 ± 11.4	50/0	SLE-DAI	3.41 ± 3.96	NP
Song et al.^39^	2013	China	Plasma	ELISA	CC	6	30	32.4 [16–55]	25/5	30	31.5 [13–56]	26/4	1997ACR	4.68 ± 0.87	15.33 ± 3.79
Mohsen et al.^38^	2013	Egypt	Serum	ELISA	CS	6	15	35.92 ± 10.6	12/3	72	34.0 ± 11.9	60/12	1997ACR	5.3 ± 4.9	23.9 ± 11.7
Shimizu et al.^36^	2012	Japan	Serum	ELISA	CC	5	32	37 ± 19	NP	45	41 ± 11	40/5	1982ACR	3.49 ± 1.19	NP
Fouad et al.^40^	2014	Egypt	Serum	ELISA	CS	8	50	26.8 ± 8.1	50/0	50	27.9 ± 7.5	50/0	SLICC	7 ± 3.5	15.7 ± 4.9
Bakry et al.^41^	2015	Egypt	Serum	ELISA	CC	8	20	25.5 ± 6.3	18/2	40	26.3 ± 7.9	35/5	1982ACR	0.8 ± 0.13	13.7 ± 4.3
Girard et al.^42^	2016	Switzerland	Serum	ELISA	CS	7	40	46 (36–55.25)	15/25	29	39 (30–47)	25/4	1982ACR	6 (3–13)	3 (0–15.3)
Jafari-Nakhjavani et al.^43^	2016	Iran	Plasma	ELISA	CS	6	50	29.48 ± 7.2	43/7	113	30.74 ± 10.49	103/10	ACR	NP	NP
Sigdel et al.^44^	2016	China	Serum	ELISA	CS	8	24	37.37 ± 9.30	21/3	49	37.4 ± 9.3	45/4	1997ACR	NP	16.1 ± 3.6
Petrackova et al.^45^	2017	Czech Republic	Serum	PEA	CS	7	23	40 [26–73]	15/8	75	40 [19–74]	66/9	1997ACR	11 [1–38]	7 [0–43]
Italiani et al.^46^	2018	Italy	Serum	ELISA	CC	6	80	NP	NP	74	39.5 (30–50)	64/10	1997ACR	10 (4–20)	NP
Mende et al.^11^	2018	Australia	Serum	ELISA	CC	7	52	36 (26.8, 44.1)	39/13	184	44.9 ± 14	167/17	1997ACR	10.2 (6, 17.2)	4 (2–6)
Umare et al.^12^	2019	India	Serum	ELISA	Cohort	6	201	29.2 ± 11	187/14	200	28 ± 10	184/16	1997ACR	2.3 ± 0.6	16.6 ± 7.9

Data are presented in the form of mean ± standard deviation, or median [range], or mean (inter quartile range).

*F/M* female versus male, *CC* case control, *CS* cross-sectional, *SLEDAI* systemic lupus erythematosus disease activity index, *NP* not provided, *ELISA* enzyme linked immunosorbent assay, *PEA* proximity extension immunoassay, *ECL* electrochemiluminescence essay, *ACR* American College of Rheumatology diagnostic standard, *SLICC* Systemic Lupus International Collaborating Clinics, *SLE-DAI* Toronto SLE Disease Activity Index.

### SLE patients had high levels of circulating IL-18 compared with healthy controls

**Figure 2:**
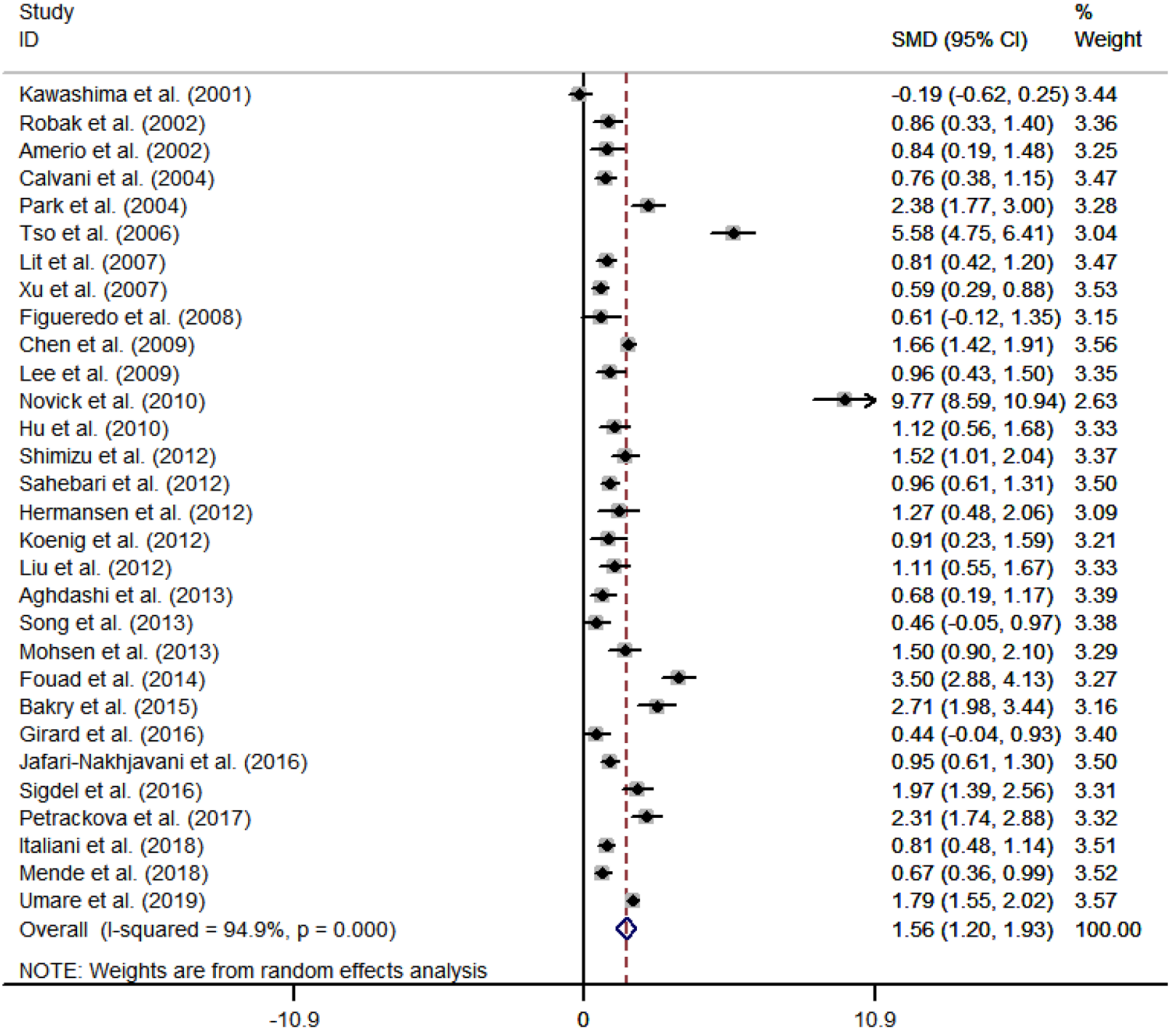
Forrest plot of the standard mean variance (SMD) for the levels of circulating IL-18 in systematic lupus erythematosus patients and healthy controls using a random-effect model. (StataCorp. 2013. Stata Statistical Software: Release 13. College Station, TX: StataCorp LP. www.stata.com).

We pooled data from altogether 30 studies after computing SMD and its 95% CI for each of them. Results displayed that SLE patients showed significantly higher levels of circulating IL-18 in comparison with healthy controls (SMD = 1.56, 95% CI [1.20–1.93]). Heterogeneity was obvious (I^2^ = 94.9%, *p* < 0.01) and therefore, a random-effects model was applied (Fig. 2). Subgroup analyses based on age, ethnicity, sample type, disease duration, SLEDAI score, testing method and main meta-analysis results were summarized in Table 2. SLE patients had higher IL-18 level both in plasma and serum (SMD = 1.45, 95% CI [0.51–2.38]; SMD = 1.59, 95% CI [1.19–1.99]). Besides, Asian, White, Arab and mixed SLE patients all exhibited elevated IL-18 level in comparison with healthy controls except for Latin American patients (SMD = 1.48, 95% CI [0.97–1.98]; SMD = 1.68, 95% CI [0.94–2.42]; SMD = 2.57, 95% CI [1.36–3.78]; SMD = 0.71, 95% CI [0.46–0.95]; SMD = 0.61, 95% CI [− 0.12 to 1.35]). Increased IL-18 had also been observed in SLE patients from all age groups and whose disease duration was more than or less than five years (SMD = 2.20, 95% CI [1.40–3.00]; SMD = 1.28, 95% CI [0.89–1.67]; SMD = 2.35, 95% CI [1.13–3.57]; SMD = 1.72, 95% CI [1.11–2.34]; SMD = 1.24, 95% CI [0.73–1.75]). What’s more, if SLEDAI scores for SLE patients were over five which underlay more active disease severity, the level of IL-18 escalated higher than normal people (SMD = 1.46, 95% CI [0.47–2.44]; SMD = 1.42, 95% CI [0.77–2.08]; SMD = 1.76, 95% CI [1.29–2.23]). However, it was not applicable to patients with ≤ 5 SLEDA score (SMD = 2.19, 95% CI [− 0.09 to 4.47]). Different testing method groups, namely ELISA, Cytokine Multiplex Assay, ECL and PEA all exhibited the same higher level in SLE patients (SMD = 1.31, 95% CI [1.00–1.63]; SMD = 1.07, 95% CI [0.55–1.58]; SMD = 9.77, 95% CI [8.59–10.94]; SMD = 2.31, 95% CI [1.74–2.88]).

**Table 2:** Subgroup meta-analysis of circulating interleukin-18 in systemic lupus erythematosus patients.

Stratification group	N	SMD (95% CI)	Heterogeneity test			
			Q	*p* value	I^2^ (%)	Egger's test *p* value
**Sample type**						
Plasma	6	1.45 (0.51, 2.38)	129.31	< 0.001	96.1	0.197
Serum	24	1.59 (1.19, 1.99)	423.34	< 0.001	94.6	0.179
**Combined**	30	1.56 (1.20, 1.93)	567.66	< 0.001	94.9	0.059
**Age (y)**						
< 30	5	2.20 (1.40, 3.00)	44.1	< 0.001	90.9	0.549
30–40	15	1.28 (0.89, 1.67)	163.14	< 0.001	91.4	0.492
≥ 40	7	2.35 (1.13, 3.57)	238.34	< 0.001	97.5	0.032
**Combined**	27	1.70 (1.31, 2.08)	500.51	< 0.001	94.8	0.062
**Disease duration (y)**						
≤ 5	7	1.24 (0.73, 1.75)	54.68	< 0.001	89.0	0.442
> 5	11	1.72 (1.11, 2.34)	213.83	< 0.001	95.3	0.163
**Combined**	18	1.52 (1.12, 1.93)	268.59	< 0.001	93.7	0.424
**SLEDAI score**						
≤ 5	3	2.19 (− 0.09, 4.47)	125.37	< 0.001	98.4	0.365
5–10	3	1.46 (0.47, 2.44)	18.07	< 0.001	88.9	0.645
10–15	4	1.42 (0.77, 2.08)	18.43	< 0.001	83.7	0.227
> 15	8	1.76 (1.29, 2.23)	68.01	< 0.001	89.7	0.839
**Combined**	18	1.69 (1.28, 2.10)	257.00	< 0.001	93.4	0.258
**Ethnicity**						
Asian	13	1.48 (0.97, 1.98)	229.74	< 0.001	94.8	0.692
White	11	1.68 (0.94, 2.42)	243.37	< 0.001	95.9	0.047
Arab	3	2.57 (1.36, 3.78)	20.8	< 0.001	90.4	0.771
Mixed	2	0.71 (0.46, 0.95)	0.13	0.718	0	NA
Latin American	1	0.61 (− 0.12, 1.35)	NA	NA	NA	NA
**Combined**	30	1.56 (1.20, 1.93)	567.66	< 0.001	94.9	0.059
**Testing method**						
ELISA	26	1.31 (1.00, 1.63)	347.09	< 0.001	92.8	0.355
Cytokine Multiplex Assay	2	1.07 (0.55, 1.58)	0.46	0.499	0	NA
ECL	1	9.77 (8.59, 10.94)	NA	NA	NA	NA
PEA	1	2.31 (1.74, 2.88)	NA	NA	NA	NA
**Combined**	30	1.56 (1.20, 1.93)	567.66	< 0.001	94.9	0.059

*N* number, *y* year, *SMD* standard mean difference, *SLEDAI* Systemic lupus erythematosus disease activity index, *NA* not available, *ELISA* enzyme linked immunosorbent assay, *PEA* proximity extension immunoassay, *ECL* electrochemiluminescence essay.

### Sensitivity analysis and meta-regression

**Figure 3:**
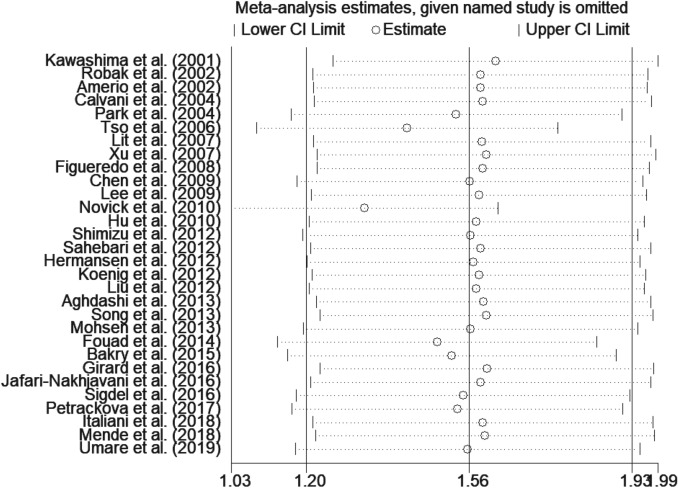
Sensitivity analysis of the pooled standard mean variance (SMD). (StataCorp. 2013. Stata Statistical Software: Release 13. College Station, TX: StataCorp LP. www.stata.com).

Since the heterogeneity of the association between circulating IL-18 and SLE was pronounced, thus additional sensitivity test was done (Fig. 3). There were no conspicuous alternations detected when removing one of the included studies and pooling the rest. On the whole, it indicated that the primary results of this meta-analysis were relatively robust.

To further explore the source of heterogeneity, meta-regression was utilized and continuous variables including publication year, mean age of SLE groups, group size, quality score, disease duration and SLEDAI score were seen as co-variables at a time, yet none of them had shown statistical significance (*p* > 0.05). Also, we transformed categorical variables into dummy variables and performed meta-regressions with sample type, study design and ethnicity. With these elements contained in the regression model separately, the main meta results hadn’t changed with *p* value all above 0.05.

### Publication bias

**Figure 4:**
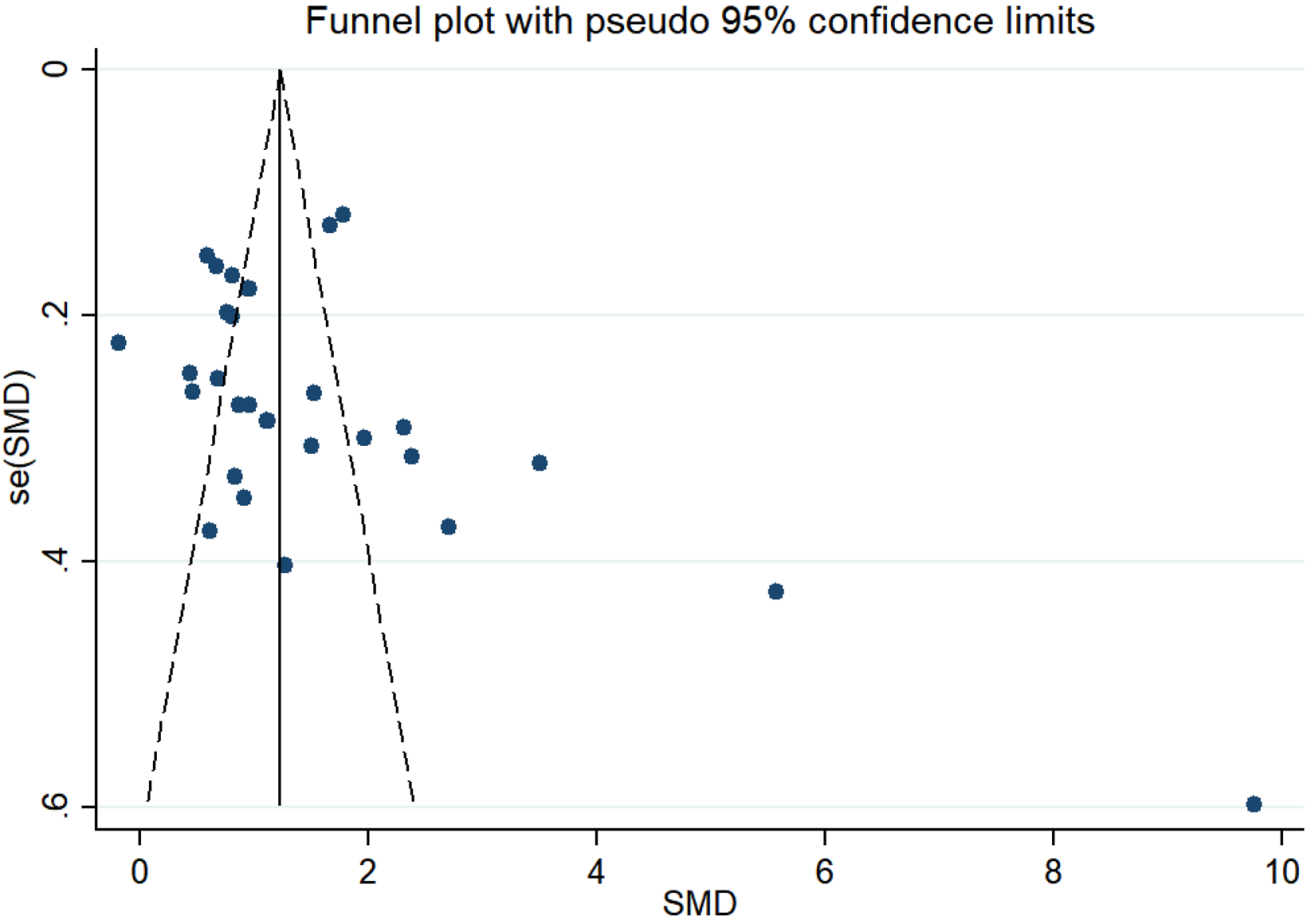
Funnel plot to assess potential publication bias. (StataCorp. 2013. Stata Statistical Software: Release 13. College Station, TX: StataCorp LP. www.stata.com).

None statistically remarkable bias was detected in Egger’s linear regression test (*p* = 0.059), Trim and fill method was conducted since asymmetry was still presented in the funnel plot (Fig. 4). After filling in the three hypothetical missing studies, the adjusted SMD remained significant (SMD = 1.22, 95% CI [0.68–1.56], *p* < 0.01) which was in accordance with previous outcomes (Supplementary Fig. S3).

## Discussion

This meta-analysis clarified the association for circulating IL-18 and SLE by containing 30 published correlated studies of 1968 SLE patients and 1439 healthy controls from 18 regions. After processing all extracted relevant data, we found that compared with healthy people, the circulating level of IL-18 was much higher in SLE patients, which indicated the underlying role of IL-18 in SLE pathogenesis. What’s more, such an association was likewise significant when included studies were subdivided based on sample type, disease duration, patient age, and testing method. In patients whose SLEDAI score was less than five or from Latin America, the IL-18 didn’t elevate significantly. Sensitivity analysis presented that the primary result remained unchanged when any of these included studies were exempted. Publication bias was not detected in Egger’s linear regression test with *p* value above 0.05. In all, the conclusion that circulating IL-18 is closely connected with SLE can be drawn and it can be utilized as a new immune marker to identify SLE patients.

Conclusions drawn from our meta-analysis were in an agreement with most of the included individual studies which strengthened the reliability. In addition, several recent studies have reported correlations between IL-18 and disease severity, organ involvement and a list of classic testing biomarkers like anti-dsDNA antibody, C3, C4 and etc^32,37^. In lupus nephritis, IL-18 is also related with proteinuria and renal activity and elevates earlier than the occurrence of proteinuria and high disease severity score, indicating that IL-18 has the chance to be used as a predictive marker to distinguish diverse stages of LN, even in subclinical stage^41^. These updated findings can help broaden the clinical applications of IL-18 and enrich its comprehensive significance in SLE.

In our study, the increased circulating IL-18 was detected in both Asian, White, mixed, and Arab group but not in Latin American population. But since only one study was from Brazil of Latin American and the size of this study was relatively small, only 16 SLE patients and 14 healthy controls, the conclusion was still in need of confirmation by a well-designed large-scale study^27^. In the Asian and White group, conclusions were more reliable to some extent since over ten studies were contained in each subgroup. What’s more, subgroup analysis had revealed that the correlation with IL-18 and SLE was significant in patients who had > 5 SLEDAI score, but not in the group where the SLEDAI score was below five. The results indicated that IL-18 has certain connections with SLE disease activity and may serve as a biomarker to identify the severity of SLE. Another study had proven that serum IL-18 differed in SLEDAI-2k ≤ 4 group versus the SLEDAI-2k > 4 group. The longitudinal analysis had confirmed that variation of serum IL-18 level from baseline was associated with SLEDAI-2K change after following up^11^. In lupus nephritis, a significant correlation had been detected between serum IL-18 and SLEDAI, renal activity score and activity index^38^.

This analysis also has several limitations that need to be carefully considered. First, the study population of several included studies is relatively small or from local medical clinics which would reduce the quality and explanatory power in our meta-analysis. Second, heterogeneity is a general problem that most of the meta-analyses may encounter. In our analysis, we had chosen a random-effect model to pool the data and detailed subgroup analyses and sensitivity analyses were performed. The still existing heterogeneity may generate bias and unreliability to some extent. Besides, due to the shortcomings of meta-analysis, our study was unable to answer the causative relationship between SLE and IL-18 and longitude data were lacked. Still, there are abundant advantages that assure the meaning of this meta-analysis. First, we searched altogether five databases to retrieve published studies as much as we can. Second, thirty studies from 18 separate regions including 1986 SLE patients and 1439 healthy controls were gathered, which was, by and large, geographically and numerically comprehensive. Besides, to better illustrate our results, subgroup analyses based on consequential clinical dimensions were done and sensitivity analysis guaranteed the reliability.

## Conclusion

To sum up, this meta-analysis found that elevated circulating IL-18 was observed in SLE patients compared with normal healthy individuals which was influenced by SLEDAI score and ethnicity, but not sample type, age group, disease duration and testing method. In the future, more well-designed or large-scale studies ought to be arranged to strengthen and explore the functions of IL-18 not only in the mechanism but also in clinical application of SLE.

## Supplementary information

Supplementary information.

## Data Availability

All data generated or analyzed during this study are included in this published article (and its Supplementary Information files).
